# Microbial Diversity for Biotechnology 2014

**DOI:** 10.1155/2015/604264

**Published:** 2015-07-28

**Authors:** George Tsiamis, Ameur Cherif, Dimitrios Karpouzas, Spyridon Ntougias

**Affiliations:** ^1^Department of Environmental and Natural Resources Management, University of Patras, 2 Seferi Street, 30100 Agrinio, Greece; ^2^LR11-ES31, Biotechnology and Bio-Geo Resources Valorization, ISBST, BiotechPole Sidi Thabet, University of Manouba, 2020 Ariana, Tunisia; ^3^Department of Biochemistry and Biotechnology, University of Thessaly, Ploutonos 26 and Aiolou Street, 41221 Larisa, Greece; ^4^Department of Environmental Engineering, Democritus University of Thrace, Vasilissis Sofias 12, 67100 Xanthi, Greece

The focus of this special issue is the characterization of the microbial diversity and the development of potential biotechnological applications. Despite the great importance of microbes, only a small fraction has been able to be cultivated in the laboratory. It is now clear that molecular techniques have played a significant role in the detection and unravelling of the enormous microbial diversity. Omic technologies like genomics, metagenomics, and single cell genomics enabled the characterization of a vast and unexplored portion of the microbial diversity.

Human life and microbes are highly coupled since key biogeochemical cycles like carbon, nitrogen, and sulfur are microbially dependent. Even the photosynthetic capacity on Earth is microbial dependent and not plant based. In humans, the gut symbionts assist in food digestion, toxin breakdown, and boosting the immune system. In an attempt to understand the unculturable fraction programs like the Earth Microbiome Project (EMP), the International Census of Marine Microbes, and the Human Microbiome Project have been launched. The next step is to use this boundless and fascinating resource for the discovery of new genes and enzymes for use in biotechnology.

This special issue includes nine research articles describing the microbial diversity and the deployment of new technologies that can lead to prospective biotechnological applications.

D. H. Green et al. studied the bacterial diversity associated with the coccolithophorid algae* Emiliania huxleyi* and* Coccolithus pelagicus* f.* braarudii*. Coccolithophores make significant contributions to oceanic carbon cycling and atmospheric CO_2_ regulation, but despite their importance the bacterial diversity associated with these algae has not been explored from ecological or biotechnological point of view. Culture-dependent and independent analyses indicated that the bacterial diversity was spread across five phyla.* Alphaproteobacteria*,* Gammaproteobacteria*, and* Bacteroidetes* were the most dominant, followed by* Actinobacteria* and* Acidobacteria*.

G. Merlino et al. present the characterization of the bacterial community developed in anaerobic microcosms after biostimulation with lactate from a groundwater polluted with 1,2-dichloroethane (1,2-DCA). Molecular approaches targeting conserved regions of known reductive dehalogenase genes identified four novel variants that have been associated with the reductive dechlorination of 1,2-DCA.

H. Lin et al. examined the microbial community structure and pathogen occurrence in urban faucet biofilms in South China using microstructure analysis and 454 pyrosequencing.* Proteobacteria* were found to be the most predominant group in all biofilms samples. Interestingly, some potential pathogens (e.g.,* Legionellales* and* Enterobacteriales*) and corrosive microorganisms were also found in these biofilms.

H. Xu et al. examined the heterologous expression of cellulases in* Thermotoga* sp. isolated from* Caldicellulosiruptor saccharolyticus*. Although the vectors were lost from the* Thermotoga* strain after three consecutive transfers, all three recombinant enzymes were successfully expressed rendering the host with increased endo- and/or exoglucanase activities. This work demonstrated the feasibility of genetically engineering* Thermotoga* spp. for efficient cellulose utilization.

E. G. Di Domenico et al. explored the anaerobic digestate as feed in H-type MFC reactors: one with a graphite anode preconditioned with* Geobacter sulfurreducens* and the other with an unconditioned graphite anode. Their results indicated that MFCs can be used to recover anammox bacteria from natural sources. This opens the possibility of using anaerobic digester for the simultaneous nitrogen removal and electricity generation.

G. A. Silva-Castro et al. studied the precipitation of calcium carbonate and calcium sulphate by isolating bacteria from seawater and real brine.* Bacillus* and* Virgibacillus* strains were able to form calcium carbonate minerals, which precipitated as calcite and aragonite crystals. Further characterization of these bacterial strains is of great importance for the bioremediation of CO_2_ and calcium capture in certain environments.

A. Krasowska et al. isolated and characterized at molecular level the bacteriophages of* Bacillus subtilis*. The studied phages were from the Myoviridae and the Siphoviridae family. The host range, pH and temperature resistance, adsorption rate, latent time, and phage burst size were determined. These findings can be used in industry since* Bacillus* spp. are very persistent strains.

M. Nampally et al. searched for novel chitin and chitosan modifying enzymes isolated from soil microorganisms with more than ten years of history of chitin and chitosan exposure. After screening for chitinase and chitosanase isoenzymes, eight bacterial strains and twenty fungal isolates were found to be chitinolytic and/or chitosanolytic.

R. A. Amer et al. examined the physical, chemical, and microbiological features of hydrocarbon and heavy metals contaminated sediments collected at El-Max Bay (Egypt). Following a culture-dependent approach fifty bacterial strains were isolated and were identified as hydrocarbon-degrading bacteria involved in different stages of biodegradation. The El-Max sediment represents a promising reservoir of novel bacterial strains adapted to high hydrocarbon contamination loads.

These papers highlight the importance of the study and characterization of the hidden microbial diversity and the vast potential that exists in order to transform this basic knowledge to applied knowledge with the assistance of biotechnology. New developments in the field of omic approaches will certainly propel our ability to understand and further exploit microbial diversity. In this regard, these papers provide a glimpse into the future of this field.

